# Serum sST2 levels predict severe exacerbation of asthma

**DOI:** 10.1186/s12931-018-0872-2

**Published:** 2018-09-03

**Authors:** Masato Watanabe, Keitaro Nakamoto, Toshiya Inui, Mitsuru Sada, Kojiro Honda, Masaki Tamura, Yukari Ogawa, Takuma Yokoyama, Takeshi Saraya, Daisuke Kurai, Haruyuki Ishii, Hajime Takizawa

**Affiliations:** Department of Respiratory Medicine, Kyorin University School of Medicine, 6-20-3 Sinkawa, Mitaka-city, Tokyo, 181-8612 Japan

**Keywords:** IL-33, ST2L, Biomarker

## Abstract

**Background:**

Neutrophilic inflammation is associated with poorly controlled asthma. Serum levels of sST2, a soluble IL-33 receptor, increase in neutrophilic lung diseases. We hypothesized that high serum sST2 levels in stable asthmatics are a predictor for exacerbation within a short duration.

**Methods:**

This prospective observational study evaluated the serum sST2 levels of 104 asthmatic patients who were treated by a lung disease specialist with follow-ups for 3 months.

**Results:**

High serum sST2 levels (> 18 ng/ml) predicted severe asthma exacerbation within 3 months. Serum sST2 levels correlated positively with asthma severity (treatment step), airway H_2_O_2_ levels, and serum IL-8 levels. High serum sST2 levels and blood neutrophilia (> 6000 /μl) were independent predictors of exacerbation. We defined a post-hoc exacerbation-risk score combining high serum sST2 level and blood neutrophilia, which stratified patients into four groups. The score predicted exacerbation-risk with an area under curve of 0.91 in the receiver operating characteristic curve analysis. Patients with the highest scores had the most severe phenotype, with 85.7% showing exacerbation, airflow limitation, and corticosteroid-insensitivity.

**Conclusions:**

High serum sST2 levels predicted exacerbation within the general asthmatic population and, when combined with blood neutrophil levels, provided an exacerbation-risk score that was an accurate predictor of exacerbation occurring within 3 months.

**Electronic supplementary material:**

The online version of this article (10.1186/s12931-018-0872-2) contains supplementary material, which is available to authorized users.

## Background

Bronchial asthma is a clinical syndrome characterized by chronic airway inflammation, respiratory symptoms, airflow limitation, and bronchial hypersensitivity [1]. Asthma has heterogeneous pathogenic causes and can be classified into different phenotypes [2, 3].

In particular, neutrophilic airway inflammation is found in patients with poorly controlled asthma [4–6]. Neutrophilic asthma is characterized by a high neutrophil count in the sputum (40–76%), along with severe asthma, corticosteroid-insensitivity, chronic airflow obstruction, and acute exacerbation [4, 5, 7–9]. Furthermore, mixed granulocytic (i.e. neutrophilic and eosinophilic) infiltration into the airway causes the greatest disease burden and airflow limitation in asthmatic patients [8, 9]. Patients with uncontrolled asthma commonly use oral corticosteroid, which may further augment airway neutrophilia. Peripheral blood neutrophilia is another predictor for exacerbation and poor control of asthma [10], which may be related to oral corticosteroids usage. Importantly, the association between neutrophil counts in sputum and peripheral blood is very weak [5]. Combined, these results indicate that asthma related to neutrophilic inflammation can be further classified into subtypes, e.g. airway- or blood-dominance and combined. However, no current serum biomarkers efficiently stratify neutrophil-related asthma into subgroups.

Soluble suppression of tumorigenicity 2 (sST2) is a decoy receptor for interleukin (IL)-33 [11, 12]. IL-33 is released from bronchial epithelial cells and lung blood vessels after exposure to allergic antigens [13] and necrosis [14]. IL-33 causes eosinophilic inflammation and Th2 cytokine production in the lungs [14–17] and is involved in arising asthma [17, 18]. In contrast, sST2 is released from bronchial epithelial cells and lung blood vessels by stimulation with proinflammatory cytokines, toll-like receptor (TLR) ligands, and Th2 cytokines [11, 19]. Serum sST2 levels are markedly increased when neutrophilic inflammation is present (i.e. in pneumonia [20], chronic obstructive lung disease [COPD], [21] and sepsis [22]). In patients with atopic asthma, serum sST2 levels are elevated during exacerbation and are associated with the severity of the asthma attack [23]. These indicate that the IL-33/sST2 balance affects granulocyte counts in the airway, and that high serum sST2 levels in asthmatics are associated with exacerbation occurrences.

We hypothesized that high serum sST2 levels in stable asthmatics are a predictor for exacerbation within a short period of time. We also expected that serum sST2 levels would stratify neutrophil-related asthma into subgroups. To address these, we conducted a prospective observational study, in which we recruited 104 asthmatic patients, measured serum sST2 levels, followed-up for 3 months, and evaluated whether serum sST2 levels predicted exacerbation. We also explored the characteristics of patients with high serum sST2 levels, and found that serum sST2 levels and blood neutrophilia were independent predictors of asthma exacerbation. In a post-hoc decision, we defined an asthma exacerbation-risk score based on high serum sST2 levels and blood neutrophilia. Patients that were positive for both were at extremely high exacerbation risk (85.7%), indicating that serum sST2 levels and blood neutrophil counts can classify poorly controlled asthma into clinically relevant subgroups.

## Methods

### Study design

This was a prospective observational study approved by the institutional review board at Kyorin University (approvals no. 161 and no. 523). All patients gave written informed consent before participating; patients underwent blood tests, lung function tests, and fractional exhaled nitric oxide (FeNO) tests, followed by obtaining exhaled breath condensates (EBC). Afterward, the patients were treated by lung disease specialists in accordance with the guidelines of the Global Initiative for Asthma (GINA) 2015 [24] and were followed-up for 3 months. The primary endpoint was severe exacerbation: defined as worsening asthma that required hospital admission or an emergency room visit.

### Patients

We recruited patients with asthma who visited the Department of Respiratory Medicine, Kyorin University Hospital from January 2013 through November 2015. All patients were diagnosed with asthma and assessed for treatment step by a lung disease specialist according to the criteria of the GINA 2015. No patients had experienced any exacerbation of asthma for at least 4 weeks prior to participating in the study.

### Laboratory testing and cytokine measurements

Serum levels of sST2, IL-6, IL-8, IL-33, and IL-5 were quantified using Quantikine ELISA kits (R&D Systems, MN, USA) according to the manufacturer’s instructions. EBC was obtained with an RTube (Respiratory Research, TX, USA). Serum and EBC H_2_O_2_ levels were measured using a d-ROMs test® (WISMERLL, Tokyo, Japan). The FeNO level was measured using a NIOX MINO® device (Aerocrine AB, Solna, Sweden) according to the manufacturer’s instructions and the American Thoracic Society-guidelines [25].

### Statistics

Normality was assessed using the Shapiro-Wilk test. Data are shown as mean ± SD and median (interquartile range) for parametric and non-parametric data, respectively. For parametric data, two or more groups were compared using the Student’s *t*-test or one-way ANOVA, respectively. For non-parametric data, two or more groups were compared using the Mann-Whitney or Kruskal-Wallis tests, respectively. Categorical data were compared using a Chi-square test or Fisher’s Exact test. Multiple comparisons for parametric and non-parametric data were performed using the Dunnett test and the Steel test, respectively. For all correlations, the Spearman’s correlation coefficient was used. Using the receiver operating characteristic (ROC) curve analysis, the area under curve (AUC) and cut-off values (determined by the Youden index) were calculated. Hazard ratios were calculated using a Cox-regression analysis. Power analysis was performed with an alpha value of 0.05. Statistical analyses were performed using SPSS statistics version 19.0.0 (IBM, New York, USA), SigmaProt version 11.0 (Systat Software Inc., Illinois, USA), and the free website provided by Osaka University (Osaka, Japan; http://www.gen-info.osaka-u.ac.jp/MEPHAS/steel.html).

## Results

### Characteristics of the patients

A total of 104 asthmatic patients were enrolled. During the three-month follow-up period, 11 patients experienced exacerbation (at-risk), whereas 93 patients did not (stable). Triggers of exacerbation were natural worsening of asthma in 10 patients and infection in one. At-risk patients showed poorer control of asthma, a higher treatment step, higher percentage of oral corticosteroid usage, higher WBC and blood neutrophil counts, lower blood eosinophil counts, higher serum H_2_O_2_ and IL-6 levels, and lower percentages of predicted vital capacity (%VC) and forced vital capacity (%FVC) than stable patients (Table 1). Smoking status, serum IgE levels and FeNO did not differ between groups. No patient was treated with biotherapy.

**Table 1:** Patient characteristics

	Stable	At-risk	*P*-value*†
	(*N* = 93)	(*N* = 11)	
Age, mean (SD)	52.7 (15.4)	51.7 (19.4)	0.852
Sex, M: F, *n* (%)	39 (42.0): 54 (58.0)	3 (27.3): 8 (72.7)	0.540
BMI, median (IQR)	22.9 (20.6–26.9)	24.6 (21.8–30.1)	0.089
Smoking, *n* (%)			
Current-smoker	8 (8.6)	2 (18.2)	0.454
Ex-smoker	27 (29.0)	4 (36.4)	
Never-smoker	58 (62.4)	5 (45.4)	
Asthma control, *n* (%)			
Well controlled	29 (31.2)	1 (9.1)	**< 0.001**
Partially controlled	51 (54.8)	2 (18.2)	
Uncontrolled	13 (14.0)	8 (72.7)	
Treatment step, *n* (%)			
1	4 (4.3)	0 (0.0)	**0.008**
2	14 (15.1)	0 (0.0)	
3	20 (21.5)	0 (0.0)	
4	49 (52.7)	7 (63.6)	
5	6 (6.4)	4 (36.4)	
Oral CS use, *n* (%)	6 (6.5)	4 (36.4)	**0.011**
Laboratory tests			
WBC (/μL), median (IQR)	6300 (5000–7200)	10,500 (6700–12,100)	**< 0.001**
Neutrophil (/μL), median (IQR)	3618 (2745–4772)	8159 (3953–9196)	**< 0.001**
Eosinophil (/μL), median (IQR)	201 (128–334)	79 (32–303)	**0.029**
CRP (mg/dl), median (IQR)	0.1 (0–0.2)	0.1 (0–0.5)	0.208
IgE (IU/ml), median (IQR)	188 (45–533)	185 (12–1539)	0.841
IL-8 (pg/ml), median (IQR)	13.1 (10.6–16.9)	16.0 (9.6–22.6)	0.612
IL-6 (pg/ml), median (IQR)	1.0 (0.5–1.7)	2.4 (1.2–3.7)	**0.035**
Serum H_2_O_2_ (U.CARR), median (IQR)	336 (302–380)	379 (350–421)	**0.025**
FeNO (ppb), median (IQR)	24 (16–42)	16 (11–99)	0.302
EBC H_2_O_2_ (U.CARR), median (IQR)	0.5 (0.1–1.0)	0.5 (0.2–0.6)	0.687
Lung function tests			
VC (L), median (IQR)	3.1 (2.5–4.0)	2.8 (2.5–4.0)	0.067
%VC (%), mean (SD)	107.4 (16.1)	94.5 (18.9)	**0.016**
FVC (L), median (IQR)	3.0 (2.5–3.9)	2.5 (1.9–3.6)	0.067
%FVC (%), mean (SD)	98.8 (15.6)	87.4 (18.7)	**0.027**
FEV_1_ (L), median (IQR)	2.3 (1.8–2.9)	1.7 (1.3–2.9)	0.104
%FEV_1_ (%), mean (SD)	89.6 (19.1)	80.1 (26.1)	0.135
FEV_1_/FVC (%), median (IQR)	75.2 (68.4–81)	73.6 (64.3–83.4)	0.958

*P-values for parametric, non-parametric, and categorical data were calculated using the Student’s *t*-test, Mann-Whitney test, and Chi-square test, respectively

†Bold letters, *P* < 0.05

*BMI* body mass index, *Oral CS* oral corticosteroid, *WBC* white blood cells, *FeNO* fractional exhaled nitric oxide, *EBC* exhaled breath condensate, *SD* standard deviation, *IQR* interquartile range

### Serum sST2 levels and high-risk exacerbation patients

**Fig. 1:**
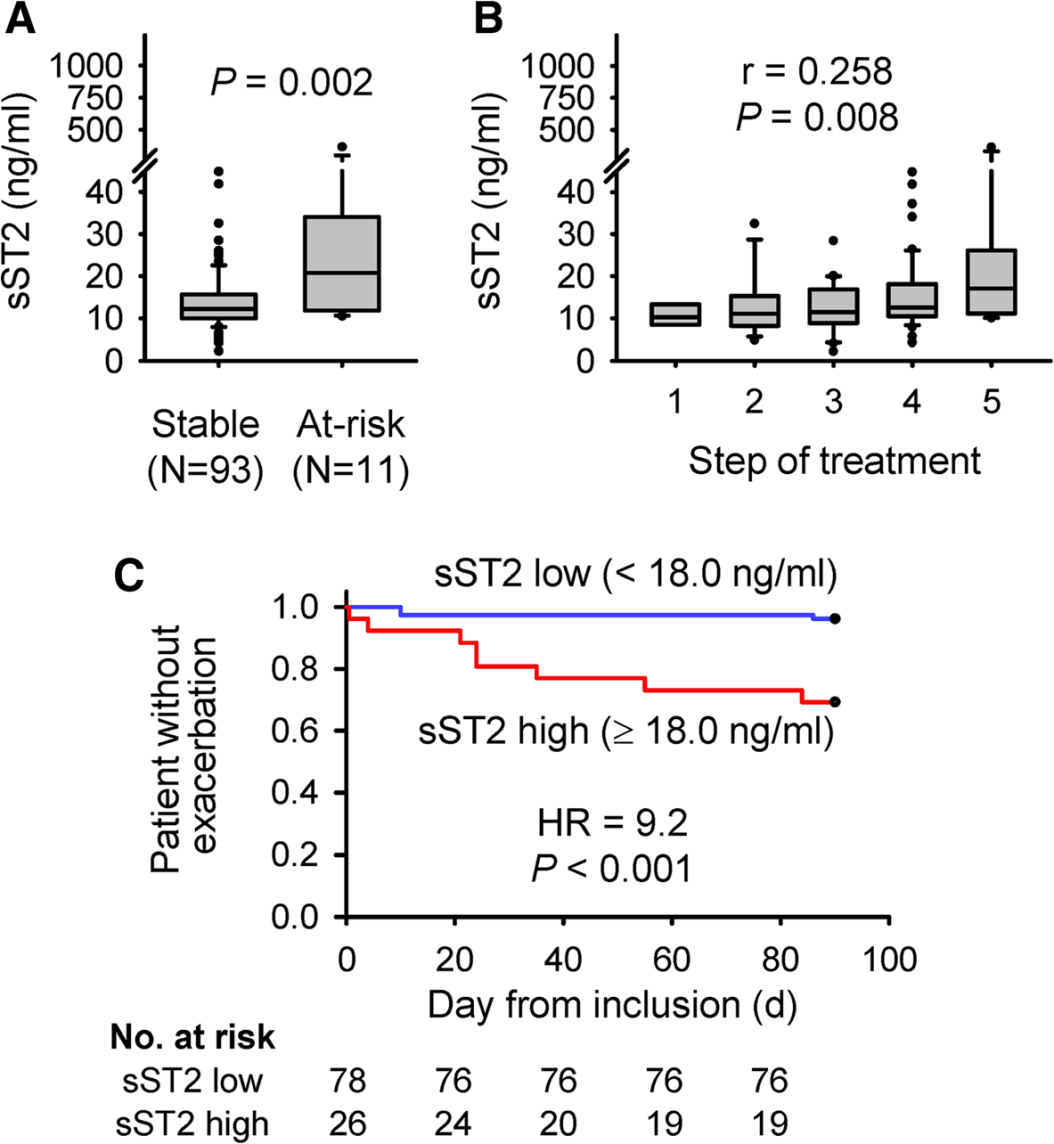
Serum sST2 levels and asthma exacerbation risk. **a** Serum sST2 levels were higher in patients whose asthma was exacerbated within 3 months (at-risk) than those without exacerbation (stable). **b** Serum sST2 levels correlated positively with the treatment steps defined in the GINA guidelines (2015). **c** Survival analysis comparing durations until exacerbation between patients with high and low serum sST2 levels. **a**–**c** *p*-values were calculated using the Mann-Whitney test (**a**), Spearman’s rank correlation (**b**), and Cox regression analysis (**c**)

At-risk patients showed higher serum sST2 levels than stable patients (*p* = 0.002, Fig. 1a). A patient with extremely high sST2 levels (364 ng/ml) was exacerbated due to infection within 1 day. Serum sST2 levels correlated positively with treatment step (*r* = 0.258, *p* = 0.008, Fig. 1b). Serum sST2 levels predicted at-risk patients with an AUC (95% CI) of 0.79 (0.63–0.94) in a ROC curve analysis. A cut-off value of 18.0 ng/ml was diagnostic of patients at high risk of exacerbation (sensitivity 0.73, specificity 0.81), with a hazard ratio (95% confidence interval) of 9.2 (2.4–34.7, *P* = 0.001; Fig. 1c). Importantly, a power analysis showed that high serum sST2 levels predicted at-risk patients with a power of 0.983 (α = 0.05, *N* = 104, *p* < 0.001), confirming that this study has a sufficient sample size.

These findings demonstrate that serum sST2 levels correlate positively with asthma severity and can diagnose patients at high risk of exacerbation.

### Serum sST2 levels and neutrophilic inflammation

To explore the characteristics of patients with high serum sST2 levels, we evaluated the correlation between serum sST2 levels and clinical parameters. Serum sST2 levels correlated positively with serum IL-8 levels and airway oxidative stress levels (EBC H_2_O_2_) (Table 2). These findings indicate that serum sST2 levels reflect the extent of airway inflammation and systemic chemotaxis activity. On the other hand, blood neutrophil counts correlated positively with serum CRP, IL-6, oxidative stress (H_2_O_2_) levels, and BMI, and negatively with blood eosinophil (%), serum IgE levels, FeNO levels, and %VC (Table 2). These results suggest that blood neutrophil counts are associated with the extent of systemic inflammation, blood oxidative stress, and obesity, and are strongly associated with neutrophilic inflammation but not eosinophilic or atopic inflammation. Unexpectedly, serum sST2 levels and blood neutrophil counts did not correlate (Table 2). Furthermore, we confirmed that high serum sST2 levels and blood neutrophilia (6000/μl) were independent predictors for asthma exacerbation (Table 3). Importantly, serum sST2 levels were not affected by oral corticosteroid usage, although WBC and blood neutrophil counts were elevated in asthmatics who used regular oral corticosteroid (Additional file 1: Figure S1). We also measured serum IL-5 and IL-33 levels in 40 subjects as preliminary experiments, but the levels were under the detection limit in all 40 subjects.

**Table 2:** Correlations between serum sST2 levels and clinical parameters

	Serum sST2 level (*N* = 104)		Blood neutrophil count (*N* = 104)	
	*r*	*P*-value*	*r*	*P*-value*
EBC H_2_O_2_ level^a^	0.281	**0.011**	0.074	0.513
FeNO	0.045	0.653	−0.306	**0.002**
Serum IL-8 level	0.328	**0.001**	0.011	0.915
Serum IL-6 level	0.089	0.370	0.384	**0.001**
Serum CRP level	0.002	0.981	0.305	**0.002**
Serum IgE level	0.046	0.643	−0.241	**0.014**
Serum H_2_O_2_ level	−0.099	0.318	0.214	**0.029**
BMI	0.185	0.060	0.205	**0.037**
WBC	0.138	0.164	0.901	**< 0.001**
Blood neutrophil count	0.129	0.191	ND	ND
Blood eosinophil count	−0.162	0.100	−0.187	0.057
Blood neutrophil (%)	0.107	0.278	0.794	< 0.001*
Blood eosinophil (%)	−0.206	0.036*	− 0.409	< 0.001*

*Bold letters, *P* < 0.05 calculated using Spearman rank correlation

^a^*N* = 81

*R* = correlation coefficient, *FeNO* fractional exhaled nitric oxide, *EBC* exhaled breath condensate, *WBC* white blood cell

**Table 3:** Multivariate analysis for predicting the risk of asthma exacerbation

	Univariate		Multivariate^a^	
	Hazard ratio (95% CI)	*P*-value	Hazard ratio (95% CI)	*P*-value*
High serum sST2 level (> 18 ng/ml)	9.2 (2.4–34.7)	0.001	5.5 (1.4–21.5)	0.015
Blood neutrophilia^b^ (> 6000 μl)	26.6 (7.9–100.9)	< 0.001	18.9 (4.8–74.4)	< 0.001

*P-values were calculated using Cox proportional hazard analysis

^a^Correlation coefficients: high serum sST2 level, 1.70; blood neutrophilia, 2.94, respectively

^b^The cut-off value for defining blood neutrophilia was optimized using the receiver operating characteristic curve analysis and calculating the Youden index

*CI* confidence interval

Taken together, high serum sST2 levels and blood neutrophilia reflect airway and systemic inflammation, respectively, and are independent predictors for asthma exacerbation.

### Definition of exacerbation-risk score and the score-based phenotypes

**Fig. 2:**
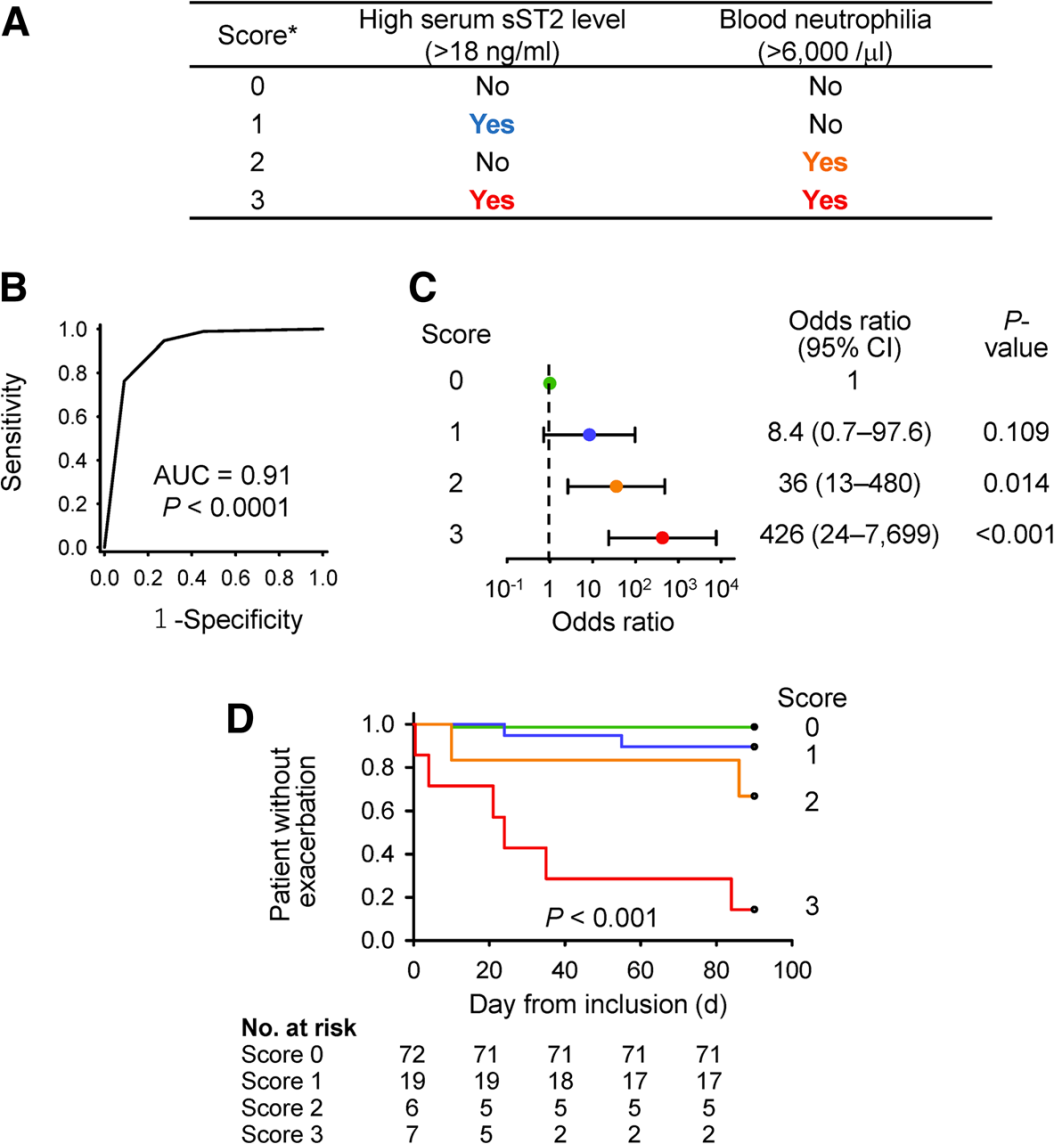
The exacerbation-risk score and its accuracy. **a** Definition of the exacerbation-risk score. Scores of 1 and 2 were based on the coefficients calculated in Table 3 (high serum sST2 level, 1.70; blood neutrophilia, 2.94) (**b**) The exacerbation-risk score predicted asthma exacerbation with an AUC (95% CI) of 0.91 (0.79–1.02) (*P* < 0.001) in the ROC curve analysis. **c** Odds ratios predicting exacerbation risk for scores of 1, 2, and 3 as compared with a score of 0. **d** Survival analysis comparing time to exacerbation among patients with scores ranging from 0 to 3. *P* < 0.05: score 0 vs. 2, score 0 vs. 3, and score 1 vs. 2. (B–D) *P*-values were calculated using a ROC curve analysis (**b**), Fisher’s Exact test (**c**), and Log-Rank test (**d**). CI, confidence interval

With a post-hoc decision, we defined an exacerbation-risk score for asthma based on serum sST2 levels and blood neutrophil count (Fig. 2a). The exacerbation-risk score predicted asthma worsening with an AUC of 0.91 (*P* < 0.001, Fig. 2b) in the ROC curve analysis. The odds ratios of scores 1 to 3 for predicting exacerbation were 8.4 (*P* = 0.109), 35.5 (*P* = 0.014), and 426 (*P* < 0.001), respectively (Fig. 2c). A survival curve analysis also showed that patients with scores of 2 and 3 were at higher risk of exacerbation than patients with scores of 0 (Fig. 2d). The sensitivities and specificities of each score for predicting exacerbation are shown in Additional file 1: Table S1. Importantly, a power analysis showed that high exacerbation-risk score predicted at-risk patients with a power of 1.000 (α = 0.05, *N* = 104, *P* < 0.001), confirming that this study has a sufficient sample size.

Finally, we assessed the characteristics of patients with each score (Table 4). Patients with a score of 3, with both high serum sST2 levels and blood neutrophilia, showed the most severe phenotype. Six out of seven patients (85.7%) experienced exacerbation; their asthma was mostly uncontrolled rather than in intensive treatment (a high treatment step), with airflow limitation and a lower percentage of eosinophil in WBC. Patients with a score of 2, with blood neutrophilia alone, showed a 33.3% exacerbation rate, identical treatment step, and better asthma control compared to patients with a score of 3, and preserved lung function, indicating that they were relatively reactive to corticosteroids. Patients with a score of 1, with high serum sST2 levels alone, showed a 10.5% of exacerbation rate, higher serum IL-8 levels, and a slightly higher treatment step than those with a score of 0. Patients with a score of 0, without serum sST2 level elevation nor blood neutrophilia, were at the lowest exacerbation risk (1.4% exacerbation rate).

**Table 4:** Characteristics of patients classified with the exacerbation-risk score

	Exacerbation-risk score				*P*-value*
	0	1	2	3	
	(*N* = 72)	(*N* = 19)	(*N* = 6)	(*N* = 7)	
Patients with exacerbation of asthma, *n* (%)	1 (1.4)	2 (10.5)	2 (33.3)	6 (85.7)	**< 0.001**
Age, mean (SD)	54.1 ± 14.6	56.1 ± 17.3	47.2 ± 20.0	59.7 ± 18.4	0.321
Sex, M: F, *n* (%)	24 (33.8): 48 (66.7)	13 (68.4): 6 (31.6)	2 (33.3): 4 (66.7)	3 (42.9): 4 (57.1)	0.050
BMI, median (IQR)	22.9 (20.6–27.4)	23.1 (21.5–26.7)	23.1 (19.6–28.6)	27.9 (21.8–30.1)	0.538
Smoking, *n* (%)					
Current smoker	8 (11.1)	1 (5.3)	0 (0)	1 (28.6)	0.527
Ex-smoker	19 (26.4)	6 (31.6)	4 (66.7)	2 (14.3)	
Never-smoker	45 (62.5)	12 (63.2)	2 (33.3)	4 (57.1)	
Asthma control, *n* (%)					
Well controlled	20 (27.8)	7 (36.8)	3 (50.0)	0 (0)	**< 0.001**
Partially controlled	42 (58.3)	9 (47.4)	1 (16.7)	1 (14.3)	
Uncontrolled	10 (13.9)	3 (15.8)	2 (33.3)	6 (85.7)	
Treatment step, *n* (%)					
1	4 (5.5)	0 (0.0)	0 (0.0)	0 (0.0)	**0.005**
2	11 (15.3)	1 (5.3)	1 (16.7)	1 (14.2)	
3	16 (22.2)	4 (21.0)	0 (0.0)	0 (0.0)	
4	39 (54.2)	12 (63.2)	2 (33.3)	3 (42.9)	
5	2 (2.8)	2 (10.5)	3 (50.0)	3 (42.9)	
sST2 (ng/ml), median (IQR)	11.0 (8.8–13.0)	21.1 (18.8–28.4)	11.8 (11.4–14.3)	26.4 (20.8–34.0)	**< 0.001**
WBC (/μL), median (IQR)	6200 (5225–6800)	5900 (4700–7200)	10,250 (9000–12,350)	10,500 (9700–12,100)	**< 0.001**
Neutrophil (/μL), median (IQR)	3496 (2786–4268)	3553 (2248–4650)	6333 (6117–10,381)	8159 (7497–9196)	**< 0.001**
Eosinophil (/μL), median (IQR)	199 (133–335)	201 (45–307)	229 (75–677)	87 (32–303)	0.273
Eosinophil (%), median (IQR)	3.7 (2.1–6.2)	3.4 (0.9–4.9)	2.4 (0.6–7.4)	**1.0 (0.3–2.5) †**	**0.045**
CRP (mg/dl), median (IQR)	0.1 (0–0.2)	0.0 (0–0.)	0.1 (0–0.4)	0.1 (0.1–0.5)	0.195
IgE (IU/ml), median (IQR)	201 (45–684)	236 (104–330)	60 (8–843)	73 (3–2656)	0.432
IL-8 (pg/ml), median (IQR)	13.0 (10.5–15.8)	**21.9 (9.7–31.0) †**	11.7 (7.9–19.8)	16.0 (11.0–25.2)	0.063
IL-6 (pg/ml), median (IQR)	1.0 (0.5–1.8)	0.9 (0.5–1.9)	2.5 (1.2–8.0)	1.8 (0.3–3.7)	0.143
Serum H_2_O_2_ (U. CARR), median (IQR)	345 (315–401)	322 (294–356)	341 (304–390)	383 (336–390)	0.182
FeNO (ppm), median (IQR)	23.5 (16.0–38.3)	24.0 (15.0–63.0)	19.0 (13.3–49.8)	16.0 (11.0–121.0)	0.892
EBC H_2_O_2_ (U. CARR) ^a^	0.4 (0.0–1.0)	0.5 (0.2–1.1)	0.4 (0.0–0.6)	0.6 (0.3–0.7)	0.751
VC (L), median (IQR)	2.9 (2.5–3.8)	3.7 (2.6–4.4)	3.7 (3.3–3.8)	**2.3 (2.0–2.9) †**	0.079
%VC (%), mean (SD)	106.7 (45.6)	107.9 (19.5)	114.0 (13.0)	**87.3 (14.2) ‡**	**0.014**
FVC (L), median (IQR)	2.9 (2.4–3.7)	3.7 (2.5–4.4)	3.6 (3.2–3.8)	**2.2 (1.8–2.9) †**	0.087
%FVC (%), mean (SD)	98.6 (14.6)	97.1 (15.4)	107.1 (15.4)	**79.4 (12.1) ‡**	**0.009**
FEV_1_ (L), median (IQR)	2.3 (1.8–2.7)	2.5 (1.4–3.1)	2.8 (2.0–3.0)	1.6 (1.3–1.9)	0.142
%FEV_1_ (%), mean (SD)	90.1 (17.8)	86.5 (23.5)	97.7 (24.3)	**71.1 (22.3) ‡**	0.063
FEV_1_/FVC (%), median (IQR)	76.0 (69.2–81.0)	70.3 (67.6–79.8)	79.4 (66.0–82.0)	73.6 (64.0–83.6)	0.550

*P-values for parametric, non-parametric, and categorical data were calculated using a one-way ANOVA test, Kruskal-Wallis test, and Chi-square test, respectively

†*P* < 0.05 vs. a score of 0, calculated using the Steel test

‡*P* < 0.05 vs. a score of 0, calculated using the Dunnett test

*†‡ Bold letters, *P* < 0.05

^a^A total of 81 patients (scores of 0, 1, 2, and 3: *N* = 56, 17, 4, and 4, respectively) were evaluated

*BMI* body mass index, *WBC* white blood cells, *FeNO* fractional exhaled nitric oxide, *EBC* exhaled breath condensate, *SD* standard deviation, *IQR* interquartile range

Taken together, high serum sST2 levels (greater than 18 ng/ml) are reasonable predictor of exacerbation and, when combined with blood neutrophilia, provided an exacerbation risk score that is an accurate predictor of exacerbation and which classifies poorly-controlled asthma into subgroups.

## Discussion

The present study demonstrated that serum sST2 levels serve as a biomarker for the disease severity of asthma and predicted exacerbation risk within 3 months. Serum sST2 levels reflected the extent of airway oxidative stress and serum chemotaxis activity, whereas peripheral neutrophil counts reflected systemic inflammatory response, blood oxidative stress, and obesity. High serum sST2 levels and peripheral blood neutrophilia independently predicted asthma exacerbation, and a double positive was suggestive of an extremely high risk of worsening asthma, corticosteroid-resistance, and airflow limitation.

High serum sST2 levels serve as biomarker for asthma severity. Oshikawa and colleagues evaluated atopic asthma patients and reported that serum sST2 levels during exacerbation were higher than these at steady state and were associated with asthma severity (i.e. peak flow volume and arterial blood CO_2_ partial pressure) [23]. We assessed asthmatic patients who were recruited irrespective of atopic state and demonstrated that serum sST2 levels at steady state also correlated with disease severity (i.e. treatment step) and that serum sST2 levels greater than 18 ng/ml were a reasonable predictor for exacerbation within 3 months. Taken together, serum sST2 levels in asthmatics correlate with severity at steady state and during exacerbation, and are a reasonable predictor for exacerbation regardless of atopic state.

sST2 is produced in the lungs [11, 19]. Multiple types of human lung cells (i.e. bronchial cells, alveolar cells, and vascular endothelial cells) release sST2 in vitro, which can be enhanced by stimulation with proinflammatory cytokines, lipopolysaccharide, and Th2 cytokines [11, 19]. Serum (or plasma) sST2 levels were elevated in patients with neutrophilic (i.e. bacterial pneumonia [20] and COPD [21]) and eosinophilic (i.e. acute eosinophilic pneumonia [26]) lung disease. Serum sST2 levels correlated with disease severity in pneumonia patients [20]. These suggest that serum sST2 levels reflect the extent of airway inflammation. In addition, airway H_2_O_2_ levels correlate with sputum granulocyte (i.e. neutrophil and eosinophil) counts in asthmatic patients [27]. We demonstrated the positive correlation between serum sST2 levels and airway H_2_O_2_ levels in asthmatics, which also indicated an association between high serum sST2 levels and the presence of airway inflammation. Taken together, serum sST2 levels reflect the extent of neutrophil and/or eosinophil infiltration in lungs.

Neutrophilic inflammation is associated with the development of uncontrolled asthma. Airway neutrophilic inflammation is related to severe asthma; airway mixed-granulocytic infiltration (i.e. both neutrophils and eosinophils) appeared to cause the greatest disease burden in asthmatics [8, 9]. In addition to sputum neutrophilia, peripheral blood neutrophilia was also a risk factor for asthma worsening [10]. However, blood neutrophil counts showed only a low association with sputum neutrophil count [5, 28], suggesting that neutrophilic inflammation-related asthma could be further classified (e.g. into subgroups of airway-dominant, blood-dominant, and mixed neutrophilia). We classified asthmatics into four subtypes using blood neutrophilia (related to systemic inflammation) and high serum sST2 levels (associated with airway inflammation), which provided an accurate predictor for exacerbation (the exacerbation-risk score). These support the concept that neutrophil-related asthma can be further stratified into subgroups that reflect phenotypic severity. Although further investigation regarding the relationship between sputum granulocyte counts (i.e. neutrophil and eosinophil counts) and serum sST2 levels are needed, our observations indicate that sST2 is a potential phenotypic marker for uncontrolled asthma.

IL-33 is related to eosinophilic asthma: it is the nuclear-associated cytokine of IL-1, [29] which is released from allergen-exposed cells [13]. Exogenous administration of IL-33 causes a massive infiltration of eosinophils and Th2 cytokine production [14–16]. In addition, IL-33 stimulates type 2 innate lymphoid cells to produce IL-5 and -13 resulting in eosinophilia and goblet cell hyperplasia, respectively [16]. Genome-wide association studies (GWASs) have demonstrated that *IL-33* and *IL1RL1* (encoding the IL-33 receptor) variants were associated with arising asthma [17, 18] and blood eosinophilia [17]. The *IL-1RL1* variant was also associated with arising neutrophilic asthma whereas the *IL-33* variant was not [17]. Further, some *IL1RL1* variants enhance sST2 production, as demonstrated by human plasma analysis and cell culture experiments [30]. Our data show that high serum sST2 levels are related to low blood eosinophil (%), which was also associated with less airway eosinophilic inflammation [22]. Taken together, an interaction between IL-33, the cell surface, and soluble IL-33 receptors (ST2L and sST2, respectively) regulates the airway granulocyte (i.e. neutrophil and eosinophil) counts that potentially influence asthma phenotypes.

The sST2/IL-33 axis regulates neutrophilic inflammation in the lungs [29, 31]. In a mouse model of influenza virus infection, IL-33 synthesis increased in the lungs [32]. Similarly, in a mouse COPD exacerbation model caused by influenza virus infection, nasal administration of IL-33 enhanced neutrophil infiltration into lungs, which was attenuated by sST2 administration [33]. IL-33, known as alarmine, [34] is released from necrotized cells [11]. IL-33 is processed by neutrophil elastase from a less-active full-length form to a highly-activated cleaved form [35]. IL-33 restored a decrease in neutrophil chemotaxis that resulted from TLR stimulation [31]. Thus, IL-33 augments neutrophilic airway inflammation in certain situations, such as when viral infections accompany necrotic tissue damage. Importantly, inflamed epithelial cells release an increased amount of sST2 [11, 19]. This attenuates biologically IL-33-induced neutrophilic inflammation and serves clinically as a biomarker for lung inflammation. Taken together, the sST2/IL-33 balance is regulated in the lungs, controlling neutrophilic inflammation and subsequent tissue damage, and serving as a biomarker for airway inflammation.

This study has some limitations. First, it is a relatively small-scale and single-centre study, thus, our findings require further confirmation by a larger study. Second, we did not evaluate sputum cells nor serum IL-33 levels. The relationship among serum sST2 and IL-33 levels, blood neutrophil counts, and asthma phenotypes should also be evaluated with sputum cell counts in future studies. However, our study provides novel diagnostic methods that counter these limitations. Firstly, serum sST2 levels greater than 18 ng/ml were a reasonable predictor for exacerbation within 3 months. Secondly, high serum sST2 levels and blood neutrophilia provided an exacerbation-risk score that can classify neutrophil-related asthma into subgroups where the subgroup with the highest score had an 85.7% exacerbation rate.

## Conclusion

High serum sST2 levels predict exacerbation in the general asthmatic population and, when combined with blood neutrophil levels, provide an exacerbation-risk score that is a better predictor of exacerbation than serum sST2 levels alone.

## Additional file

Additional file 1: **Table S1.** The exacerbation scores and their predictive values for worsening asthma. **Figure S1.** Relationship between oral corticosteroid usage and biomarkers. (DOCX 387 kb)
