# Supplementary material for: Serum sST2 levels predict severe exacerbation of asthma
Source: Respir Res. 2018 Sep 3;19:169. doi: 10.1186/s12931-018-0872-2 (PMC6126416; doi:10.1186/s12931-018-0872-2)
Supplement: Supplementary file 1 — Table S1. The exacerbation scores and their predictive values for worsening asthma. Figure S1. Relationship between oral corticosteroid usage and biomarkers. (DOCX 387 kb) [file 12931_2018_872_MOESM1_ESM.docx]

**Supplemental Data**

Serum sST2 levels predict severe exacerbation of asthma

Authors:

Masato Watanabe MD, PhD^1^, Keitaro Nakamoto MD^1^, Toshiya Inui MD^1^, Mitsuru Sada MD^1^, Kojiro Honda^1^, Masaki Tamura, MD^1^, Yukari Ogawa, MD^1^, Takuma Yokoyama MD^1^, Takeshi Saraya MD, PhD^1^, Daisuke Kurai MD, PhD^1^, Haruyuki Ishii MD, PhD^1^, Hajime Takizawa MD, PhD^1^

Institution:

1. Department of Respiratory Medicine, Kyorin University School of Medicine, Tokyo, 181-8166, Japan

Corresponding Author:

Masato Watanabe, MD, PhD

Department of Respiratory Medicine, Kyorin University School of Medicine, 6-20-3 Sinkawa, Mitaka-city, Tokyo, 181-8612, Japan

Phone: +81-422-47-5511

E-mail: [masato@ks.kyorin-u.ac.jp](mailto:masato@ks.kyorin-u.ac.jp)

## Methods

## Statistics

Two groups were compared using the Mann-Whitney U test. Using receiver operating characteristic (ROC) curve analysis, the area under curve (AUC) with cut-off values and corresponding sensitivity, specificity, and negative and positive likelihood ratios were calculated. Statistical analyses were performed using SPSS statistics version 19.0.0 (IBM, New York, USA), SigmaProt version 11.0 (Systat Software Inc., Illinois, USA), and GraphPad Prism version 7 (GraphPad Software Inc, California USA).

## Table S1 The exacerbation scores and their predictive values for worsening asthma.

| Cut off value | Sensitivity  (95% CI) | Specificity  (95% CI) | Positive  LR | Negative  LR |
| --- | --- | --- | --- | --- |
| 0.5 | 0.91 (0.59–1.00) | 0.76 (0.66–0.85) | 3.8 | 0.1 |
| 1.5 | 0.73 (0.39–0.94) | 0.95 (0.88–0.98) | 13.5 | 0.3 |
| 2.5 | 0.55 (0.23–0.83) | 0.99 (0.94–1.00) | 59.5 | 0.5 |

CI = confidence interval, LR = likelihood ratio.

**Figure S1 Relationship between oral corticosteroid usage and biomarkers.**

(A) Serum sST2 levels, (B) WBC, and (C) blood neutrophil counts were compared in asthmatics with or without oral corticosteroid usage (n = 10 and 94, respectively). Horizontal and error bars show median and interquartile ranges, respectively.
